# Exploring Lawyer–Client Interaction: A Qualitative Study of Positive Lawyer Characteristics

**DOI:** 10.1007/s12207-012-9120-0

**Published:** 2012-05-08

**Authors:** Nieke A. Elbers, Kiliaan A. P. C. van Wees, Arno J. Akkermans, Pim Cuijpers, David J. Bruinvels

**Affiliations:** 1 Faculty of Law, Department of Civil Law, VU University, Amsterdam, The Netherlands; 2 Faculty of Psychology and Education, Department of Clinical Psychology, VU University, Amsterdam, The Netherlands; 3 EMGO Institute, VU University Medical Center, Amsterdam, The Netherlands; 4 Amsterdam Interdisciplinary Center of Law and Health, VU University, De Boelelaan 1105, 1081HV Amsterdam, The Netherlands; 5 Center of Excellence of the Netherlands Society of Occupational Medicine (NVAB), Utrecht, The Netherlands; 6 Coronel Institute of Occupational Health, Academic Medical Center, Amsterdam, The Netherlands

**Keywords:** Personal injury victims, Lawyer characteristics, Client-centered lawyering, Procedural justice, Therapeutic jurisprudence

## Abstract

Personal injury victims involved in compensation processes have a worse recovery than those not involved in compensation processes. One predictor for worse recovery is lawyer engagement. As some people argue that this negative relation between lawyer engagement and recovery may be explained by lawyers’ attitude and communications to clients, it seems important to investigate lawyer–client interaction. Although procedural justice and therapeutic jurisprudence had previously discussed aspects relevant for lawyer–client interaction, the client’s perspective has been rather ignored and only few empirical studies have been conducted. In this qualitative study, 21 traffic accident victims were interviewed about their experiences with their lawyer. Five desirable characteristics for lawyers were identified: communication, empathy, decisiveness, independence, and expertise. Communication and empathy corresponded with aspects already discussed in literature, whereas decisiveness, independence and expertise had been addressed only marginally. Further qualitative and quantitative research is necessary to establish preferable lawyer characteristics and to investigate what would improve the well-being of personal injury victims during the claims settlement process.

## Introduction

Personal injury victims involved in claims settlement processes have a worse physical and psychological recovery than those who are not involved in a compensation process (Gabbe, Cameron, Williamson, Edwards, Graves, & Richardson, 2007; O'Donnell, Creamer, McFarlane, Silove, & Bryant, 2010). This hampered recovery is often explained by secondary gain (Shuman, 1994) or by secondary victimization, referring to the distress caused by the compensation process and the attitude of law professionals (Cotti, Magalhães, Pinto da Costa, & Matos, 2004). One predictor for worse recovery is lawyer involvement (Dichraff, 1993; Gun, Osti, O'Riordan, Mpelasoka, Eckerwall, & Smyth, 2005; Harris, Murgatroyd, Cameron, Young, & Solomon, 2009). Several explanations for this negative association between lawyer involvement and well-being have been proposed. It could be that people who engage a lawyer have more severe injuries or that their claims are more problematic (Dichraff, 1993). However, studies that controlled for injury severity have found a similar effect (Bernacki & Tao, 2008). Other explanations were that lawyers may implicitly encourage their clients to maintain sickness behavior because ‘going back to work will damage your case’ (Aurbach, 2011), that lawyers may inflict emotional harm to clients by communicating poorly (Schatman, 2009), or that they may not sufficiently take into account the emotional dimension and non-material needs (Akkermans, 2009).

Given the fact that a negative influence of lawyers’ attitude and communication with clients has been raised, we explored the literature on the interaction between lawyers and clients in the context of procedural justice and therapeutic jurisprudence. *Procedural justice* implies that litigant’s perception of justice is determined more by procedural aspects and the way in which a decision is reached than by the outcome itself (Thibaut & Walker, 1975). Satisfaction with judicial processes depends on whether litigants get the opportunity to participate, whether they are treated with dignity and respect, and whether they trust the decision makers (Tyler, 1992). A process is perceived to be fair if (a) rules are applied consistently across persons and time, (b) decision makers are neutral, (c) the procedure is based on accurate information, (d) appeal procedures exist, (e) all subgroups are heard, and (f) the process adheres to ethical standards (Leventhal, 1980). *Interactional justice* embodies the impact of interaction and communication on the perception of fairness (Bies & Moag, 1986). People want to be treated with dignity and respect. Bies and Moag identified four criteria for interactional justice: (a) explain the basis for the decisions, (b) be truthful and candid, (c) be respectful and polite, and (d) refrain from improper remarks or prejudicial statements. *Informational justice* requires explanations to be reasonable, timely, and specific if they are to be perceived as fair (Shapiro, Buttner, & Barry, 1994). Until now, however, these justice elements have been applied only to procedures involving a neutral decision maker (Tyler, 1992), and it is not known whether they also apply to lawyer–client interaction.

The second research area is *therapeutic jurisprudence*, a multidisciplinary approach to law (Winick, 2005). Therapeutic jurisprudence practitioners argued that lawyers should consider the ‘psycho-legal soft spots’ — legal interventions or procedures that may lead to anxiety, distress, depression, and hard or hurt feelings (Patry, Wexler, Stolle, & Tomkins, 1998). Therapeutic jurisprudence teaches lawyers the basic principles of psychology, interpersonal skills, listening, interviewing and counseling techniques, and ways of dealing with emotional issues (Sternlight & Robbennolt, 2008). Lawyers are also encouraged to involve the client in decision-making and to adhere to client-centered lawyering (Binder, Bergman, & Price, 1990; Kruse, 2006). However, therapeutic jurisprudence seems to theorize lawyer–client interaction from the lawyer’s perspective rather than the client’s point of view. Additionally, only a few empirical studies have been conducted on the topic.

Only a few studies investigating the claimant’s perspective were found in relation to lawyer–client interaction. One study interviewed a sample of male claimants in New York (Rosenthal, 1974). One-third of the claimants were dissatisfied with the professional service they received. For example, their lawyers did not prepare them for the pretrial stress, or they did not hear from their lawyers for a considerable time, or the lawyers conducted business over the telephone using a bored and patronizing tone of voice. A more recent study, in which Dutch accident victims were interviewed (Stichting De Ombudsman, 2003), mainly highlighted the lack of lawyer–client communication: a lot of the interviewees did not understand the lawyers’ letters, did not know what was going on, experienced distrust, or were afraid that their lawyers collaborated with insurance companies. Lawyers often forgot to inform their clients well, or did not explain the procedure, which made claimants lose track of their own file. Claimants were also frustrated about lawyers who lingered over their work, who did not call back, or who made mistakes in their letters.

Given the paucity of research that has been conducted, the purpose of the current study is to empirically investigate the lawyer–client relationship from the client’s perspective, specifically clients’ preferences and experiences regarding their lawyers. A qualitative research method was used, being an appropriate method for gaining knowledge on an unexplored topic and for creating a basis for further quantitative research (Strauss & Corbin, 1998). Personal injury victims were interviewed about their experiences with their lawyer. These experiences were clustered into a set of desirable characteristics for lawyers to adopt. Based on the justice and therapeutic jurisprudence literature, it was hypothesized that, in order to be satisfied, plaintiffs would want their lawyers to communicate well, to show dignity and respect, to provide information, to listen, and to involve them in decision-making. In addition, the qualitative approach allowed us to investigate whether other factors could be identified that had not yet been identified in literature.

## Method

### Participants and Procedure

Participants were recruited by Victim Support Netherlands and a personal injury law firm based in Amsterdam. The inclusion criteria were: (1) being a victim of a traffic accident, (2) being involved in claims settlement or having settled the claim no more than 2 years ago, and (3) being or having been represented by a lawyer. Recruitment continued until data saturation was reached, which means that no extra information was being obtained from the qualitative interviews.

The participants were interviewed about five topics: (a) demographic characteristics, injuries, and claims details; (b) communications between the participant and the lawyer; (c) communications between the lawyer and the insurance company; (d) the lawyer’s expertise with regard to the compensation settlement; and (e) the lawyer’s perceived strengths and weaknesses, and what qualities good lawyers should have. The interviews were semi-structured, meaning that the interviewer could deviate from the sequence of questions and could examine some themes more thoroughly than others. The interviews were conducted in Dutch, by the primary investigator (NE) and a colleague, both psychologists. Each interview took an average of 1 to 1.5 h to complete. The interviews were recorded by a voice recorder and typed out verbatim. The interviews were held between August 2008 and February 2009. The Medical Ethics Committee of the VU University Medical Center approved the study protocol.

### Analysis

The analysis consisted of labeling statements in which participants expressed their experiences and preferences with respect to their lawyer. Analyzing was done by means of open, axial and selective coding (Strauss & Corbin, 1998). In the open coding phase, the transcripts were labeled with keywords that emerged from the hypotheses. The axial coding process consisted of examining whether the labels needed to be restructured, whether sub-labels could be applied, and whether new labels had emerged. During the selective coding, all the transcripts were re-analyzed based on the refinement that had occurred during axial coding. The interviews were analyzed in duplicate by two researchers (NE and KW). During the cyclic analysis process, the two analyzers discussed their findings and, through discussion, they agreed upon the final set of labels. Analyses were conducted using the computer software program Atlas.ti (version 5.2).

## Results

### Participants

Twenty-one participants were included in the study. No new themes emerged in the final interviews so additional data was not sought after this. The study sample consisted of 12 women and nine men, with a mean age of 43 years. Eleven participants had orthopedic injuries, seven had whiplash related injuries, one had pelvic instability, and two had suffered psychological injuries. Five participants had already settled their claims, while the other 16 were still involved in claims settlement processes. The length of their involvement in the compensation process ranged from a few months to 13 years.

### Preferable Lawyer Characteristics

Preferable lawyer characteristics were derived using a process of open, axial and selective coding. In the open coding phase, we labeled four positive lawyer characteristics derived from literature: communication, information, empathy, and involvement. In the axial coding phase, it was decided not to consider ‘information’ and ‘involvement’ as separate labels, but instead to merge them with the label ‘communication’. During the interviews, it also gradually became apparent that ‘decisiveness’, ‘independence’, and ‘expertise’ were important topics. In the selective coding phase, all transcripts were re-analyzed based on five labels that had emerged in the prior phases: (a) communication, (b) empathy, (c) decisiveness, (d) independence, and (e) expertise.

#### Communication

The first aspect of communication that emerged in the qualitative analysis was involvement. Several participants appreciated being involved in the compensation process in the sense that their lawyer listened to their story and their opinions and responded to issues they had raised, either by taking action or explaining why no action was taken. Other participants, however, specifically did *not* want to be involved, either because they did not want to be bothered by the claims settlement process, or because it made them think the lawyer was not able to handle the case.

Secondly, participants wanted proper information on the compensation procedure. In other words, to be informed about what was going to happen and what they should expect during claims settlement. Some participants were displeased by being left in the dark and not being given a step-by-step overview, whereas participants who had been informed in advance about the possible scenarios and the consequences felt pleased and confident.

A third aspect of communication concerned the mode of communication, with several participants indicating that they would have preferred more face-to-face contact, at least at the start and subsequently at least once a year, rather than having only written correspondence or a conversation by telephone. Personal contact gave clients a feeling of being taken seriously and was seen as an efficient way of communicating. A few participants were indignant that their lawyer never came by and (or even) said ‘if you wanted to see me, you can come to the office’. Participants appreciated lawyers forwarding all correspondence between the lawyer and the insurance company to them. Simply forwarding letters, however, was not enough, as explanatory information also needed to be included.

Lastly, the frequency of communication was a topic of discussion. Most participants regarded a telephone call once every 2 months as a good frequency and appreciated if they were still being contacted occasionally even when nothing had happened.

#### Empathy

Empathy refers to the various experiences of our participants as to whether they felt respected and treated with dignity. Participants used words such as compassionate, understanding, interested, involved, human, accessible, personal, friendly, and nice. They indicated that they appreciated the lawyer asking how they felt, showing genuine interest, always being there for them, being able to put their mind at rest, and realizing how the injury hampered them in doing the things they value in life. One disgruntled participant would have liked to have been asked whether she managed and whether she needed help. Another participant was angry that her lawyer spoke to her in a derogatory tone.

Empathy also involved being acknowledged by the lawyer and being understood and taken seriously. One participant indicated that he really appreciated the fact that his lawyer acknowledged his feelings but at the same time took care not to lose himself in feelings of injustice against the insurance company, whereas another participant who did not feel acknowledged in his feelings of injustice, lost confidence in his lawyer.

The final finding was that the need for empathy could change during claims settlement. Some participants indicated that they needed their lawyer to be empathic at the beginning of the claims settlement, whereas later on in the process they were ready for more business-like communications.

#### Decisiveness

The interviewees appreciated having an active, decisive lawyer, as they could then step away from their claim, confident that their interests were being represented. However, many participants were burdened by feeling that they had to keep their lawyer on his/her toes and that they had to call their lawyer to get things done. Some clients did not hear from their lawyer for 1 year. According to several participants, lack of decisiveness caused their case to stagnate for unacceptably long periods of time. Some clients were bothered by their lawyer being passive and even putting a lot of work into the clients’ hands, like asking clients to put things on paper. Other participants complained that their lawyer was only active in sending bills. A couple of interviewees were convinced that their lawyer deliberately let their case come to a dead end, so that the lawyer could ‘fill his own pockets’. On the other hand, some participants believed that their lawyer acted *too* decisively in the actual settlement of the claim. These lawyers started to discuss a settlement whereas the participants did not know whether future damage was covered, and whether they would get what they were entitled to.

#### Independence

The participants’ desire for independence related to their lawyer’s attitude toward the insurance company (i.e., the opposite party). Some participants were enraged by their belief that their lawyer did not want to ‘rub the insurance company up the wrong way’, did not ‘play hard’, or ‘sacrificed their case to win a few others’. Some participants were disturbed by the fact that their lawyer obtained information via the insurance company, instead of gathering the data from its original source, as this caused their compensation process to be based on incomplete information and often also caused delay. Some clients gained confidence in their lawyers’ independence because their lawyer was seen to be open and honest about his/her attitude to the insurance company, explaining positions in the light of reoccurring professional contacts with the insurer. Independence was also required in the process of appointing medical or occupational experts to assess the plaintiff’s impairment. Some participants believed that the expert appointed by their lawyer was not truly independent but instead had connections with the insurance company.

#### Expertise

Many participants had very clear opinions on the expertise of their lawyer. Participants regarded lawyers as having good expertise if lawyers informed them about the types of damages eligible for compensation and how such compensation was assessed. According to some participants, their case was harmed by their lawyer’s lack of adequate legal experience and organizational skills needed to bring the claims settlement to a successful conclusion. Other participants were concerned by lawyers being experienced lawyers, but having little or no experience in personal injury cases. Lastly, some participants lost confidence in their lawyer because the lawyer was a ‘terrible scatterbrain’ who made an ‘incredible mess’ of the paperwork, or because the lawyer made careless mistakes in the correspondence.

## Discussion

In this study, we examined personal injury victims’ experiences with respect to their lawyers. Five preferable characteristics for lawyers were identified. The importance of good communication, of providing information, and of involving clients in decision-making had previously been discussed in the justice literature and in therapeutic jurisprudence (Binder et al., 1990; Sternlight & Robbennolt, 2008; Tyler, 1992). However, an interesting finding was that not all participants actually wanted to be involved; some indicated that they did not want to deal with the claim settlement process and preferred the ‘lawyer in control’. The need for face-to-face contact once in a while has not been examined to any great extent in the past, with only one study reporting that ‘in-person interviews offer a better opportunity than phone conversations or certainly written surveys to impress the client, build rapport, learn from the client, minimize reliance on the attorney’s prior conceptions’ (Sternlight & Robbennolt, 2008, pp. 538). Lastly, our participants preferred to be updated at least every 2 months, even if there had not been any developments. This finding is confirmed by two articles that stated a need for timely updates (Schatman, 2009; Shapiro et al., 1994).

‘Empathy’ has previously been addressed in the therapeutic jurisprudence and justice literature (Tyler, 1992; Winick, 1998), although the justice literature tends to use words such as dignity and respect. Although empathy could be considered an aspect of communication (Sternlight & Robbennolt, 2008), our participants indicated it to be very important, and it was consequently presented separately in our results. Another interesting finding was that two participants needed their lawyer to be empathic at the beginning of the claims settlement, whereas they were ready for more business-like communications later on. This could be explained by the fact that most victims are psychologically vulnerable after the accident; especially in the first few months, victims want practical help, information, and support, and they want to talk about their experience to regain a sense of control (Brom, Kleber, & Hofman, 1993).

‘Decisiveness’ had not previously been discussed as an important lawyer characteristic. This is remarkable, given that an important frustration is that the claims settlement process takes too long (Cotti et al., 2004). According to our participants, lawyers can contribute significantly to delaying or having move forward the compensation process. One article reported that lawyers should adopt a proactive approach in order to avoid or prevent litigation before it arises (Daicoff, 2006). However, the proactive approach needed to prevent legal disputes is different from the decisiveness needed to settle claims without delay, as our participants stated. Several participants in our sample were burdened by feelings that it was left up to them to ensure that their lawyer got on with his work.

‘Independence’ could be the counterpart of the problematic lawyer behavior ‘collusion with the defense counsel’, as addressed by Schatman (2009), although he discussed a rather extreme notion of misconduct and conspiracy. Another study reported that ‘clients may sometimes suspect that lawyers recommend a particular course of action because they are friends with the opposing attorney, afraid to take a case to court, afraid of hurting their own relationship with the opposing client, or seeking to aggrandize their own reputation’ (Sternlight & Robbennolt, 2008, pp. 500–501). In general, however, the literature did not consider a lack of independence to be an important problem in the same way as did our participants.

‘Expertise’ was a surprising finding because we did not expect clients to be able to assess lawyers’ legal knowledge. The findings that some participants regarded their lawyer as not being sufficiently experienced as a (personal injury) lawyer and that other lawyers were seen as making careless mistakes have some resemblance to the procedural justice element that a legal procedure should be based on accurate information (Leventhal, 1980). The finding that some participants appreciated their lawyer making it sufficiently clear what types of damages were assessed could correspond to the interactional justice element that the basis for decisions need to be explained (Bies & Moag, 1986). In contrast to the lack of support found in the literature for this factor, several participants in our study indicated that they were very concerned by their lawyer’s lack of expertise (or possibly the lawyers’ inability to communicate their expertise).

One of the strengths in our study is that we empirically confirmed factors found in the existing theories and literature about positive lawyer characteristics *and* identified new points of interest for client-centered lawyering. This qualitative study also provides an empirical basis for further quantitative research, which is needed because little empirical research has so far been performed on the topic. A limitation, however, is that we were not able to generalize the study results to plaintiffs, in general, to other types of lawyer–client interactions, or to countries with different compensation processes, such as no-fault compensation systems or litigation. Generalizability is always limited in qualitative research since qualitative researchers are looking for variation rather than representativeness (Strauss & Corbin, 1998).

Further empirical research can quantify the relevance of the five positive lawyer characteristics that emerged as important in the present qualitative study and can reveal whether there is an association between preferable lawyer characteristics and clients’ well-being. Future lawyer–client researchers could learn from health science, having a rich tradition of investigating in doctor–patient communications and the effect of verbal and nonverbal behavior on patient satisfaction, quality of life, and health (Beck, Daughtridge, & Sloane, 2002). Generally, we hope this study inspires more empirical research into the lawyer–client relationship, enhancing client satisfaction, and possibly improving the well-being of personal injury victims during the claims settlement process.

## References

[CR1] Akkermans, A. J. (2009). Reforming personal injury claims settlement: Paying more attention to emotional dimension promotes victims recovery (February 26, 2009). Available at SSRN: http://ssrn.com/abstract=1333214

[CR2] Aurbach, R. (2011). Dispute resolution as a creator of needless disability. *AMA Guides Newsletter, July/August*, 1–11.

[CR3] Beck RS, Daughtridge R, Sloane PD (2002). Physician–patient communication in the primary care office: A systematic review. The Journal of the American Board of Family Practice.

[CR4] Bernacki EJ, Tao XG (2008). The relationship between attorney involvement, claim duration, and workers' compensation costs. Journal of Occupational and Environmental Medicine.

[CR5] Bies RJ, Moag JS, Lewicki RJ, Sheppard BH, Bazerman MH (1986). Interactional justice: Communication criteria of fairness. Research on negotiation in organizations.

[CR6] Binder D, Bergman P, Price S (1990). Lawyers as counselors: A client-centered approach. New York Law School Law Review.

[CR7] Brom D, Kleber RJ, Hofman MC (1993). Victims of traffic accidents: Incidence and prevention of post-traumatic stress disorder. Journal of Clinical Psychology.

[CR8] Cotti A, Magalhães T, Pinto da Costa D, Matos E (2004). Road traffic accidents and secondary victimisation: The role of law professionals. Medicine and Law.

[CR9] Daicoff S (2006). Law as a healing profession: The 'comprehensive law movement'. Pepperdine Dispute Resolution Law Journal.

[CR10] Dichraff RM (1993). When the injured worker retains an attorney: The relationship between attorney involvement and case outcome. American Association of Occupational Health Nurses.

[CR11] Gabbe BJ, Cameron PA, Williamson OD, Edwards ER, Graves SE, Richardson MD (2007). The relationship between compensable status and long-term patient outcomes following orthopaedic trauma. Medical Journal of Australia.

[CR12] Gun RT, Osti OL, O'Riordan A, Mpelasoka F, Eckerwall CG, Smyth JF (2005). Risk factors for prolonged disability after whiplash injury: A prospective study. Spine.

[CR13] Harris IA, Murgatroyd DF, Cameron ID, Young JM, Solomon MJ (2009). The effect of compensation on health care utilisation in a trauma cohort. Medical Journal of Australia.

[CR14] Kruse KR (2006). Fortress in the sand: The plural values of client-centered representation. Clinical Law Review.

[CR15] Leventhal GS, Gergen K, Greenberg M, Willis R (1980). What should be done with equity theory? New approaches to the study of fairness in social relationships. Social exchange: Advances in theory and research.

[CR16] O'Donnell ML, Creamer MC, McFarlane AC, Silove D, Bryant RA (2010). Does access to compensation have an impact on recovery outcomes after injury?. Medical Journal of Australia.

[CR17] Patry MW, Wexler DB, Stolle DP, Tomkins AJ (1998). Better legal counseling through empirical research: Identifying psycholegal soft spots and strategies. California Western Law Review.

[CR18] Rosenthal DE (1974). Lawyer and client: Who's in charge?.

[CR19] Schatman ME (2009). Working to avoid collateral emotional harm to clients: Cases and recommendations for the personal injury attorney. Psychological Injury and Law.

[CR20] Shapiro D, Buttner EH, Barry B (1994). Explanations: What factors enhance their perceived adequacy?. Organizational Behavior and Human Decision Processes.

[CR21] Shuman DW (1994). The psychology of compensation in tort law. Kansas Law Review.

[CR22] Sternlight JR, Robbennolt J (2008). Good lawyers should be good psychologists: Insight for interviewing and counseling clients. Ohio State Journal on Dispute Resolution.

[CR23] Stichting De Ombudsman (2003). *Letselschaderegeling: Onderhandelen met het mes op tafel, of een zoektocht naar de redelijkheid [Claims settlement: Negotiating without concessions or a search for reasonableness]*. Hilversum: De Toekomst.

[CR24] Strauss AL, Corbin JM (1998). Basics of qualitative research: Techniques and procedures for developing grounded theory.

[CR25] Thibaut JW, Walker LJ (1975). Procedural justice: A psychological analysis.

[CR26] Tyler TR (1992). The psychological consequences of judicial procedures: Implications for civil commitment hearings. SMU Law Review.

[CR27] Winick BJ (1998). Client denial and resistance in the advance directive context: Reflections on how attorneys can identify and deal with a psycholegal soft spot. Psychology, Public Policy, and Law.

[CR28] Winick BJ (2005). Using therapeutic jurisprudence in teaching lawyering skills: Meeting the challenge of the new ABA standards. St. Thomas Law Review.

